# Identification of Two Clusters in Renal Pelvis Urobiome of Unilateral Stone Formers Using 2bRAD-M

**DOI:** 10.3390/microorganisms11092276

**Published:** 2023-09-10

**Authors:** Sen-Yuan Hong, Lin-Tao Miao, Jia-Qiao Zhang, Shao-Gang Wang

**Affiliations:** Department of Urology, Tongji Hospital, Tongji Medical College, Huazhong University of Science and Technology, Wuhan 430030, China

**Keywords:** urolithiasis, renal pelvis urine, urobiome, 2bRAD-M

## Abstract

Urolithiasis is a common urological disease with increasing incidence and a high recurrence rate, whose etiology is not fully understood. The application of sequencing and culturomics has revealed that urolithiasis is closely related to the urinary microbiome (urobiome), shedding new light on the pathogenesis of stone formation. In this study, we recruited 30 patients with unilateral stones and collected their renal pelvis urine from both sides. Then, we performed 2bRAD-M, a novel sequencing technique that provides precise microbial identification at the species level, to characterize the renal pelvis urobiome of unilateral stone formers in the both sides. We first found that the urobiome in the stone side could be divided into two clusters (Stone1 and Stone2) based on distance algorithms. Stone2 harbored higher microbial richness and diversity compared to Stone1. The genera *Cupriavidus* and *Sphingomonas* were overrepresented in Stone1, whereas *Acinetobacter* and *Pseudomonas* were overrepresented in Stone2. Meanwhile, differential species were identified between Stone1 and Stone2. We further constructed a random forest model to discriminate two clusters which achieved a powerful diagnostic potential. Moreover, the urobiome of the non-stone side (Control1/2) was compared with that of the stone side (Stone1/2). Stone1 and Control1 showed different microbial community distributions, while Stone2 was similar to Control2 based on diversity analysis. We also identified differentially abundant species among all groups. We assumed that there might be different mechanisms of how microbiota contribute to stone formation in two clusters. Our findings might assist in the selection of suitable medical treatments for urolithiasis.

## 1. Introduction

Urolithiasis is a common urological disease with increasing incidence and a high recurrence rate. The incidence of urolithiasis reached 6.4% in China, and the 5-year recurrence rate is estimated to be 67%, which places a huge economic burden on the healthcare system [1,2]. The most prevalent stone type is calcium oxalate (CaOx), followed by calcium phosphate (CaP), uric acid, struvite, and cystine [3]. Hypercalciuria and hyperoxaluria are major risk factors for urolithiasis, and stone formation is thought to begin with the supersaturation of stone constituents in the urine, such as calcium and oxalate [4]. However, urolithiasis is a multifactorial disease, and several factors might contribute to it, such as different genetic backgrounds and lifestyles. Thus, the pathogenesis of stone diseases remains unclear, making the development of drugs for treatment and prevention relatively stagnant.

Recently, the discovery of the urinary microbiome (urobiome) has brought a novel insight into urological diseases. The urinary tract is inhabited by a complex community of microbes, which was reported to be related with urolithiasis, urinary incontinence, urological tumors, and so on [5,6,7]. 16S rRNA gene sequencing has shown that the bladder urobiome of stone formers is distinct from that of healthy individuals [8,9]. Additionally, some bacterial species were dominant both in the bladder urine and stone homogenate from the same patient [10]. It is worth mentioning that Shen et al. have identified two clusters in the bladder urobiome of CaOx stone patients, and the two cluster have different dominant bacterial taxa and were associated with different metabolic pathways [11]. These findings hint that the urobiome might play a crucial role in urolithiasis.

Urine has a low microbial biomass compared to feces, which hinders precise and reliable microbial identification. In addition, 16S rRNA gene sequencing generally allows for taxonomic classification down to the genus level. 2bRAD-M is a novel technology for the study of microbiomes, which can provide accurate species resolution profiles in the low-biomass microbiome [12]. In this study, we recruited unilateral stone formers and collected their renal pelvis urine from both sides for 2bRAD-M sequencing. We aimed to explore whether the renal pelvis urobiome could be clustered into different groups and compare the microbial community between clusters.

## 2. Materials and Methods

### 2.1. Patient Recruitment

We recruited a total of 30 unilateral stone formers with an initial stone episode, including 21 males and 9 females, at Tongji Hospital. All patients were diagnosed via computed tomography and their clinical characteristics were collected. Stones were confirmed during percutaneous nephrolithotomy and the removed stones were sent for chemical composition analysis. The following exclusion criteria were applied to minimize the confounding factors that might affect the urobiome: urinary tract infections (UTIs), other urologic disease, history of major urological surgery, antibiotic treatment within 4 weeks, urinary catheterization within 4 weeks.

### 2.2. Sample Collection and Processing

The Ethical Review Board of Tongji Hospital, Tongji Medical College, Huazhong University of Science and Technology granted approval for the collection of renal pelvis urine, and the ethical permission number is 2021S130. All patients provided written informed consent for the utilization of their samples. To ensure the purity of the collected renal pelvis urine, a urethral catheter was employed to empty the bladder and prevent any contamination from mixed bladder urine. After that, a ureteroscope was cautiously introduced into the renal pelvis containing stone(s), while a ureteral catheter was concurrently inserted into the ureteroscope to gather urine from the renal pelvis. The ureteroscope and ureteral catheter were subsequently withdrawn upon completion of a 5 mL urine collection. Following this, the aforementioned procedure was replicated to collect urine from the alternate renal pelvis lacking stone(s), utilizing a new ureteroscope and ureteral catheter. The urine samples were promptly stored at a temperature of −80 °C within 1 h of collection. The entire process was carried out in a sterile environment.

### 2.3. DNA Extraction, Library Preparation and Sequencing

The genomic DNA was extracted using the TIANamp Micro DNA Kit (Tiangen, Beijing, China). After that, 2bRAD libraries were prepared following established protocols [12]. Initially, the genomic DNA was subjected to digestion using 4 U of BcgI restriction enzyme (NEB, Ipswich, MA, USA) at a temperature of 37 °C for a duration of 3 h. Subsequently, a ligation reaction was carried out in a reaction volume of 20 μL, comprising 10 μL of the digested product, 1 mM ATP (NEB, Ipswich, MA, USA), 0.2 μM of each library-specific adaptor (Ada1 and Ada2), 800 U of T4 DNA ligase (NEB, Ipswich, MA, USA), and 1 × T4 DNA Ligase Buffer (NEB, Ipswich, MA, USA), at a temperature of 4 °C for a duration of 16 h. The BcgI enzyme was heat inactivated at a temperature of 65 °C for a duration of 20 min. Following this, PCR amplification was conducted using the ligation products in a reaction volume of 40 μL. This reaction volume consisted of 7 μL of ligated DNA, 0.3 mM of dNTP, 0.1 μM of each primer (Primer1 and Primer2 for Illumina), 0.4 U of Phusion high-fidelity DNA polymerase (NEB, Ipswich, MA, USA), and 1× Phusion HF buffer (NEB, Ipswich, MA, USA). Each PCR reaction was carried out for 16–28 cycles, with the following temperature conditions: 98 °C for 5 s, 60 °C for 20 s, and 72 °C for 10 s. Finally, a 10 min extension at 72 °C was performed. The library products were purified with the QIAquick PCR purification kit (Qiagen, Hilden, Germany) and sequenced on Illumina HiSeq X™ Ten platform. Library construction and Illumina sequencing were performed at OE BioTech Co., Ltd., Qingdao, China.

### 2.4. Sequencing Processing and Quantitative Analysis

Enzyme reads were extracted from raw reads based on BcgI restriction enzyme recognition sites and were filtered to obtain clean reads with the following criteria: (1) removing reads where unknown bases were >8%; (2) removing low-quality reads (percentage of low-quality bases >20%. The taxonomic profiling was performed using the 2bRAD-M computational pipeline (https://github.com/shihuang047/2bRAD-M) accessed on 11 October 2021. First, clean reads were mapped against the prebuilt 2bRAD tag database (2b-Tag-DB) which contains taxa-specific BcgI-derived tags identified from 173,165 microbial genomes (including bacteria, fungi, and archaea). A minimum G score threshold of 10 was set to filter false-positive discovery and screen the candidate microbial taxa. Next, a secondary 2b-Tag-DB which only contains more specific 2bRAD tags for each candidate taxa was constructed for accurate calculation of identified taxa. All clean reads were mapped against the sample-specific 2b-Tag-DB to estimate the relative abundance of candidate taxa. The relative abundance of a given species is estimated as the ratio of the number of microbial individuals belonging to a species against the total number of individuals from known species that can be detected within a sample. Ultimately, a taxonomic abundance profile was created.

### 2.5. Bioinformatic Analysis

Samples were clustered based on 6 distance algorithms, including binary Jaccard, Bray–Curtis, Euclidean, JSD, L1, and L2 distance. The “vegan” package was applied to calculate alpha diversity indices (Chao1, Shannon, and Simpson index) which were visualized as boxplots and estimate beta diversity via distance algorithms (Bray–Curtis, binary Jaccard, and Euclidean distance) which were visualized as principal coordinate analysis (PCoA) [13]. The “VennDiagram” package was used to construct a Venn diagram and show the common and unique species between groups. Linear discriminant analysis (LDA) effect size (LEfSe) was performed to determine differential taxa between groups [14]. A random forest model was generated by the “randomForest” package using 10-fold cross-validation with 10 repeats to discriminate different clusters [15]. Then, the cross-validation error curve was generated, and the point exhibiting the lowest cross-validation error was identified as the cut-off point. The cut-off value was determined by adding the minimum error and the standard deviation (SD) at that point. Subsequently, all sets of biomarkers with errors below the cut-off value were enumerated, and the set with the fewest number of species was designated as the optimal set. To evaluate the performance of the random forest model, the “pROC” package was employed to construct the receiver operating characteristic curve (ROC) and calculate the area under the ROC curve (AUC) [16]. Finally, the most suitable species set was employed to forecast the probability of disease (POD) index for the cohort [17].

### 2.6. Statistical Analysis

SPSS (version 26) and R software (version 4.1.1) were used to perform statistical analysis. Among clinical parameters, continuous variables were expressed as mean ± SD and compared using an unpaired *t*-test, while categorical variables were expressed as percentages and compared using a Fisher’s exact test. The Wilcoxon test was applied for group comparisons of alpha diversity, and the analysis of similarities (ANOSIM) was conducted for group comparisons of beta diversity. Unpaired *t* test and paired *t* test were used for comparison of microbial communities. *p*-value < 0.05 was considered as statistically significant.

## 3. Results

### 3.1. Clustering of the Renal Pelvis Urine Samples

We obtained 7,160,308 ± 1,191,625 high-quality clean reads from each sample. After clean reads were mapped against 2b-Tag-DB, 451 species, 160 genera, 92 families, 55 orders, 24 classes, 12 phyla, and 3 kingdoms (Archaea, Bacteria and Eukaryota) were identified in all samples. We applied six distance algorithms (binary Jaccard, Bray–Curtis, Euclidean, JSD, L1, and L2 distance) to cluster the samples (Figure S1). We found that Euclidean, JSD, and L2 distance could divide the samples into three clusters with the same results (Figure S1C,D,F). Since one of the clusters only contained three samples, we eliminated those three samples from the study population. Finally, the cluster with 11 samples was referred to as Stone1 and the cluster with 16 samples was referred to as Stone2. We further focused on clinical characteristics of the Stone1 and Stone2 clusters. However, there were no significant differences between two clusters in age, gender, BMI, stone side, stone count, stone composition, and comorbidities (Table 1).

### 3.2. Biodiversity of the Renal Pelvis Urobiome between Stone1 and Stone2

**Figure 1:**
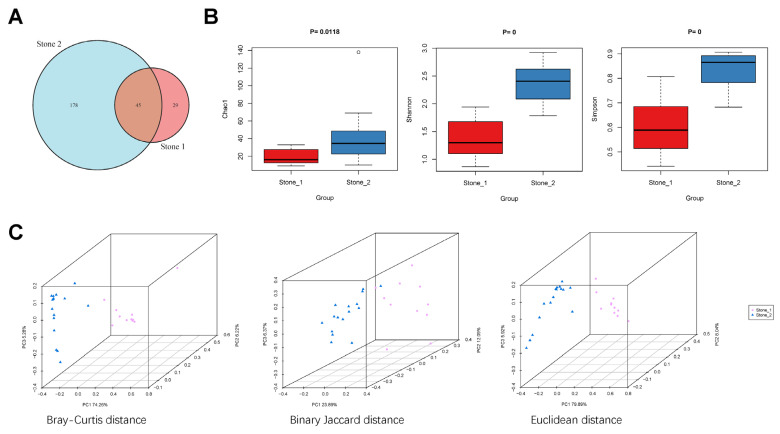
Microbial biodiversity of the renal pelvis urobiome between Stone1 and Stone2. (**A**) A Venn diagram showing the shared and unique species between two clusters. (**B**) Comparison of alpha diversity (Chao1, Shannon index, and Simpson index) between two clusters. (**C**) Comparison of beta diversity between two clusters based on Bray–Curtis distance, binary Jaccard distance, and Euclidean distance. PCoA revealed that Stone1 and Stone2 exhibited different microbial community distributions.

As shown in the Venn diagram, 45 species were shared between Stone1 and Stone2, while 29 species were unique in Stone1 and 178 species were unique in Stone2 (Figure 1A). Alpha diversity was estimated on the Chao1 index (a community richness index) and the Shannon and Simpson index (community diversity indices). The Chao1, Shannon, and Simpson indexes were higher in Stone2 than Stone1 (Figure 1B). For beta diversity, PCoA revealed that microbial composition structure was significantly different between Stone1 and Stone2 based on calculations of the Bray–Curtis distance (ANOSIM, R = 0.9863, *p* = 0.001), binary Jaccard distance (ANOSIM, R = 0.6737, *p* = 0.001), and Euclidean distance (ANOSIM, R = 0.9584, *p* = 0.001) (Figure 1C).

### 3.3. Bacterial Community Composition between Stone1 and Stone2

**Figure 2:**
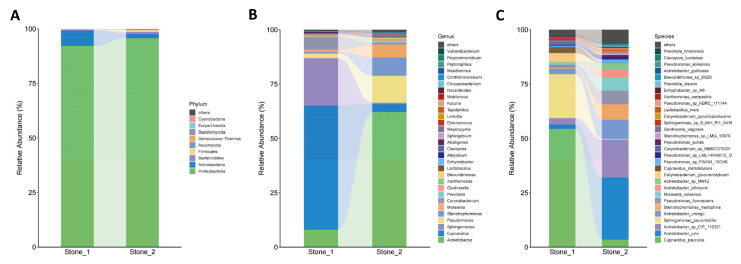
Bacterial abundance and distribution in the two clusters. The relative abundances of the bacterial phyla (**A**), the top 30 most abundant genera (**B**) and species (**C**) are represented in the barplot.

A total of 9 and 8 phyla were identified in the Stone1 and Stone2 clusters, respectively. Proteobacteria was the dominant phylum for both the groups (92.15 and 95.71%), followed by Actinobacteria (7.10 and 1.95%) and Bacteroidetes (0.29 and 0.99%) (Figure 2A). A total of 39 and 84 genera were identified in the Stone1 and Stone2 clusters, respectively. The dominant genus in Stone1 was *Cupriavidus* (57.10%), followed by *Sphingomonas* (21.80%), *Acinetobacter* (8.00%), *Corynebacterium* (5.78%), and *Pseudomonas* (2.19%), while the major genera in Stone2 were *Acinetobacter* (62.13%), *Pseudomonas* (12.40%), *Stenotrophomonas* (8.43%), *Moraxella* (5.97%), and *Cupriavidus* (3.59%) (Figure 2B). The top 30 most abundant species are shown in Figure 2C. *Cupriavidus pauculus* (54.37%), *Sphingomonas paucimobilis* (20.18%), *Corynebacterium glucuronolyticum* (4.13%), *Acinetobacter sp_CIP_110321* (2.92%), and *Cupriavidus metallidurans* (2.68%) were dominant in Stone1, whereas *Acinetobacter junii* (28.47%), *A. sp_CIP_110321* (17.44%), *Acinetobacter ursingii* (8.79%), *Stenotrophomonas maltophilia* (7.08%), and *Pseudomonas fluorescens* (6.30%) were dominant in Stone2.

### 3.4. Differential Bacterial Taxa between Stone1 and Stone2

**Figure 3:**
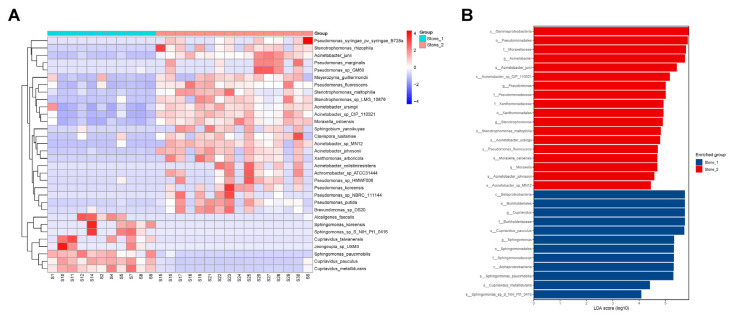
Differential microbial taxa between Stone1 and Stone2. (**A**) The heatmap shows increased abundance of 8 species and decreased abundance of 32 species in Stone1 compared to Stone2 through an unpaired *t*-test. (**B**) LEfSe exhibited 30 discriminative features (LDA score ≥ 4.0) with significant different relative abundances between Stone1 and Stone2.

The relative abundances of microbial taxa between two clusters were compared to identify the differentially represented taxa using an unpaired *t* test (Table S1). Stone1 exhibited an increased abundance of four genera and a decreased abundance of nine genera compared to Stone2. The taxa at the genus level that differentiated two clusters most were *Cupriavidus* and *Sphingomonas* in Stone1 and *Acinetobacter* and *Pseudomonas* in Stone2. We observed higher levels of 8 species and lower levels of 32 species in Stone1 compared to Stone2 (Figure 3A, Table S1). *C. pauculus* and *A. sp_CIP_110321* were the most enriched species in Stone1 and Stone2, respectively.

We also applied LEfSe to identify the differentially abundant taxa between the two clusters. LEfSe identified 30 discriminative features (LDA score ≥ 4.0) with significant different relative abundances between Stone1 and Stone2 (Figure 3B). At the species level, urobiome of Stone1 was enriched with *C. pauculus*, *S. paucimobilis*, *C. metallidurans*, and *Sphingomonas sp_S_NIH_Pt1_0416*, while the urobiome of Stone2 was enriched with *A. junii*, *A. sp_CIP_110321*, *S. maltophilia*, *A. ursingii*, *P. fluorescens*, *Moraxella osloensis*, *Acinetobacter johnsonii*, and *Acinetobacter sp_MN12*.

### 3.5. Classification of Two Clusters Using Random Forest Model

**Figure 4:**
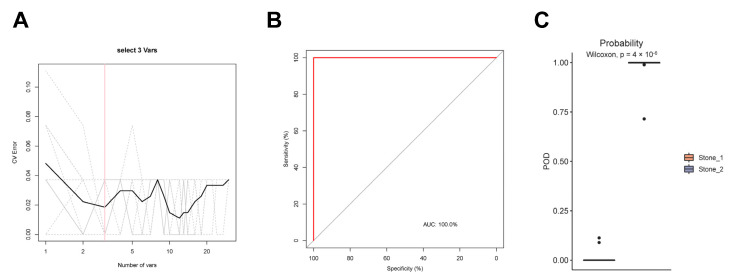
Classification of two clusters by random forest model. (**A**) The cross-validation error curve showed that three species markers were selected as the optimal marker set. (**B**) The average AUC value reached 100% between two clusters. (**C**) The POD value of Stone1 and Stone2.

Furthermore, we constructed a random forest model to identify species with diagnostic potential. The top 30 most abundant species were selected and 10-fold cross-validation with 10 repeats performed. As shown in Figure 4A, three species markers were selected as the optimal markers set to distinguish the two clusters, including *A. junii*, *C. metallidurans*, and *S. maltophilia*. ROC analysis was performed to assess the performance of the model, and the average AUC value achieved 100% between the Stone1 and Stone2 (Figure 4B). The POD value was then calculated, and the POD value was significantly increased in Stone2 compared to Stone1 (*p* < 0.001), suggesting that the POD based on microbial species markers achieved a powerful diagnostic potential for Stone2 from Stone1 (Figure 4C).

### 3.6. Biodiversity of the Renal Pelvis Urobiome between Stone1 and Control1/Stone2 and Control2

**Figure 5:**
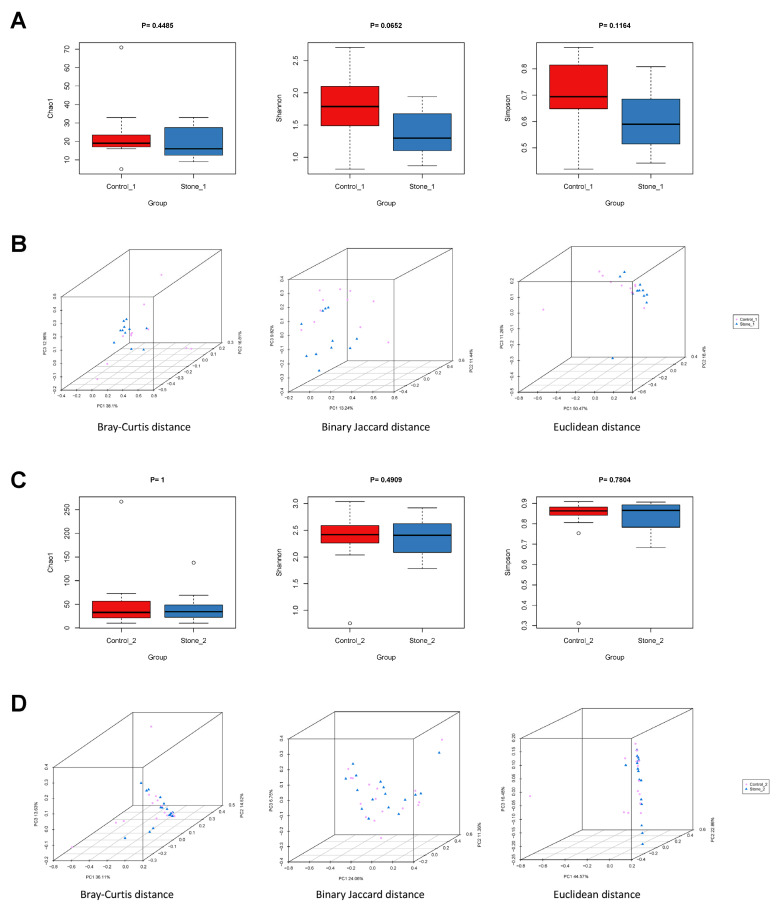
Microbial biodiversity of the renal pelvis urobiome between Stone1 and Control1/Stone2 and Control2. (**A**) Comparison of alpha diversity (Chao1, Shannon index, and Simpson index) between Stone1 and Control1. (**B**) Comparison of beta diversity between Stone1 and Control1 based on Bray–Curtis distance, binary Jaccard distance, and Euclidean distance. PCoA revealed that Stone1 and Control1 exhibited different microbial community distributions. (**C**) Comparison of alpha diversity (Chao1, Shannon index, and Simpson index) between Stone2 and Control2. (**D**) Comparison of beta diversity between Stone2 and Control2 based on Bray–Curtis distance, binary Jaccard distance, and Euclidean distance. PCoA revealed that Stone2 was similar to Control2.

We collected renal pelvis urine from both sides in the same patient, and the samples in the non-stone side compared to Stone1(2) were referred to as Control1(2). Furthermore, we explored whether the urobiome is different between Stone1 and Control1/Stone2 and Control2. For alpha diversity, the Chao1, Shannon, and Simpson indexes were not significantly different between Stone1 and Control1 or Stone2 and Control2 (Figure 5A,C). For beta diversity, PCoA showed significant difference in microbial composition structure between Stone1 and Control1 based on the Bray–Curtis distance (ANOSIM, R = 0.0876, *p* = 0.032), binary Jaccard distance (ANOSIM, R = 0.1047, *p* = 0.027), and Euclidean distance (ANOSIM, R = 0.0878, *p* = 0.043) (Figure 5B). However, no significant difference between Stone2 and Control2 was observed based on Bray–Curtis distance (ANOSIM, R = 0.0061, *p* = 0.349), binary Jaccard distance (ANOSIM, R = −0.0324, *p* = 0.837), and Euclidean distance (ANOSIM, R = −0.0075, *p* = 0.522) (Figure 5D).

### 3.7. Differential Bacterial Taxa between Stone1 and Control1/Stone2 and Control2

**Figure 6:**
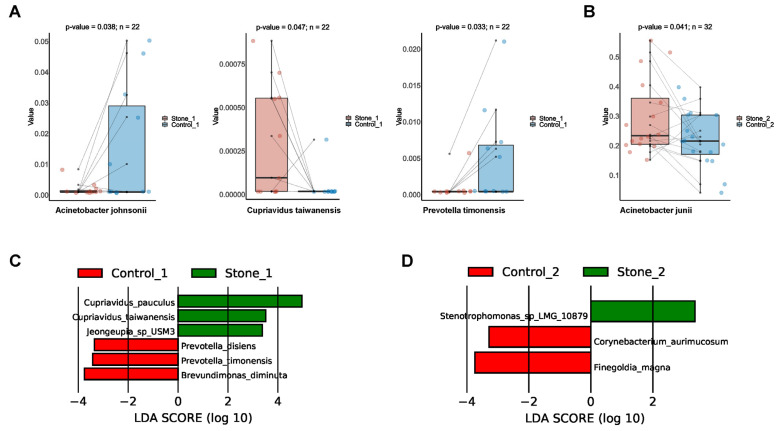
Differential microbial species between Stone1 and Control1/Stone2 and Control2. (**A**) The boxplot shows that one species was increased and two species were decreased in Stone1 compared to Control1 through a paired *t* test. (**B**) The boxplot shows one species was increased in Stone2 compared to Control2 through a paired *t* test. (**C**) LEfSe revealed six discriminative species (LDA score ≥ 2.0) with significant different relative abundances between Stone1 and Control1. (**D**) LEfSe revealed three discriminative species (LDA score ≥ 2.0) with significant different relative abundances between Stone2 and Control2.

Through paired *t* test, we found that the abundance of *Cupriavidus taiwanensis* was higher and the abundance of *A. johnsonii* and *Prevotella timonensis* was lower in Stone1 compared to Control1 (Figure 6A). Stone 2 exhibited increased abundance of *A. junii* compared to Control2 (Figure 6B). LEfSe identified six discriminative features (LDA score ≥ 2.0) whose relative abundance varied significantly between Stone1 and Control1 (Figure 6C). *C. pauculus*, *C. taiwanensis*, and *Jeongeupia sp_USM3* were enriched in Stone1, whereas *Prevotella disiens*, *P. timonensis*, and *Brevundimonas diminuta* were enriched in Control1. Likewise, three discriminative features were identified between Stone2 and Control2 via LEfSe (Figure 6D). *Stenotrophomonas sp_LMG_10879* was enriched in Stone2, while *Corynebacterium aurimucosum* and *Finegoldia magna* were enriched in Control2.

## 4. Discussion

The application of sequencing and culturomics techniques revealed that the urinary tract of stone formers harbored a distinct microbial community compared with healthy individuals, offering new insight into the pathogenesis of stone formation. However, current studies in urolithiasis all involved 16S rRNA gene sequencing to explore the urobiome, which makes it difficult to obtain precise microbial identification at the species level. Besides, most researchers collected bladder urine for the urobiome study. However, in the case of anatomic location, renal pelvis urine is more reflective of the microbiota colonizing the kidney than bladder urine. In this study, we utilized a novel species-resolved technique, 2bRAD-M, to characterize the renal pelvis urobiome in unilateral stone formers and identified two clusters (Stone1 and Stone2) in the stone side based on distance algorithms.

Beta diversity analysis verified that the renal pelvis urobiome of Stone1 and Stone2 formed distinctly different clusters in PCoA space. Through alpha diversity analysis, Stone1 showed less microbial richness and diversity than Stone2. Whether less microbial richness and diversity indicates a relative healthy status has not been determined. For example, Liu et al. reported that the bladder urobiome of healthy controls had significantly decreased microbial richness and diversity compared to that of stone formers [9], while Xie et al. found that the diversity of the bladder urobiome in male stone formers was lower than that of healthy controls [8]. Thus, the association between the richness and diversity of urinary microbial communities and disease status has not reached a consistent conclusion.

We then compared the community structure of each cluster at different taxonomic levels. The most overrepresented genera in Stone1 were *Cupriavidus* and *Sphingomonas*, and the corresponding species *C. pauculus*, *C. metallidurans*, and *S. paucimobilis* were enriched in Stone1. *C. metallidurans* has the ability to interact with metals and minerals, induce calcium carbonate precipitation and perform biomineralization [18,19]. A novel *Cupriavidus spp.* was reported to induce calcium precipitation, and its extracellular polymeric substances increased the proportion of hydroxyapatite (a naturally occurring form of CaP) in the precipitates [20]. However, the genus, *Cupriavidus*, is commonly isolated from natural environments, and their biomineralization ability has been only reported in nature, such as water and soil [18,19,20]. This is the first time that this genus was identified in human renal pelvis urine samples. Additional experimental studies would be necessary to determine whether it can induce biomineralization in the human body. Urolithiasis is considered to be a pathological biomineralization process. Randall’s plaque (RP) theory, the most widely accepted theory of CaOx stone formation, states that renal interstitial ectopic calcification, namely RPs, gradually progress subcutaneously, break through the urothelium, and serve as the attachment site of crystals in urine [21,22]. Nanoscale analysis has shown that RPs are composed of CaP and calcium carbonate [23]. Since *Cupriavidus* is capable of inducing calcium carbonate and hydroxyapatite deposits, it might play a potential causative role in the formation of RPs. *Sphingomonas* has been found to be dominant in the bladder urobiome of kidney stone patients with hypertension [9]. Moreover, the renal pelvis urobiome of male stone formers was also reported to be dominated by *Sphingomonas* [24].

The most overrepresented genera in Stone2 were *Acinetobacter* and *Pseudomonas*, and the corresponding species, *A. sp_CIP_110321*, *A. junii*, and *P. fluorescens*, were enriched in Stone2. Xie et al. have found that *Acinetobacter* is the most enriched genus in the bladder urobiome of stone formers compared to controls [8]. The increased abundance of *Acinetobacter* has also been observed in the feces of stone formers [25]. *Pseudomonas* was found to be dominant in stone homogenate by sequencing, and *Pseudomonas aeruginosa* was isolated from the same sample via enhanced quantitative urine culture [26]. Several *Pseudomonas* species, including *P. fluorescens*, have shown a strong ability to induce amorphous CaP precipitation around bacterial cells [27]. A study showed that the adhesive property of biofilms of *P. fluorescens* was increased with the presence of calcium ions [28].

The above findings shed new light on the links between urolithiasis and these bacteria. The mechanism through which bacteria in the urinary tract contribute to stone formation remains obscure. There are some plausible hypotheses based on previous research. First, bacteria can promote crystal growth and aggregation via their specific surface structures [29,30]. The contact between bacteria and CaOx crystals might be a crucial step in stone formation. Second, bacteria can adhere to the urothelium and induce the expression of stone matrix proteins and pro-inflammatory proteins [31]. Third, bacteria can change urine chemistries to create a lithogenic environment. The standard urine culture-positive stone formers showed lower urinary citrate levels than culture-negative patients, suggesting that some bacteria might produce citrate lyase and cause hypocitraturia, a major risk factor for stone formation [32]. Lastly, some bacteria have the ability to biomineralize, and they might invade and survive within the urothelium and initiate the formation of RPs. Since the two clusters were inhabited by distinct renal pelvis urobiome structures and compositions, the pathogenesis of how microbiota participate in stone formation could be different in the two clusters, and the medical treatment of the two clusters might be different. A random forest model was constructed and three species markers (*A. junii*, *C. metallidurans*, and *S. maltophilia*) were selected as the optimal markers set to distinguish the two clusters. ROC analysis and POD values further verified the powerful diagnostic potential of the model. These differentially present microbial communities may be a potentially effective tool for predicting Stone1 or Stone2, and further aid the selection of drugs for clinical treatment.

Although alpha diversity analysis revealed no differences between Stone1 and Control1 in microbial richness and diversity, beta diversity analysis showed that Stone1 and Control1 exhibited different microbial community distributions. The abundance of *C. taiwanensis*, *C. pauculus*, and *J. sp_USM3* was increased in Stone1, while the abundance of *A. johnsonii*, *P. timonensis*, *P. disiens*, and *B. diminuta* were decreased in Stone1. The urobiome structure of Stone2 was similar to that of Control2 based on alpha and beta diversity analysis. Stone2 showed increased abundance of *A. junii* and *S. sp_LMG_10879* and decreased abundance of *C. aurimucosum* and *F. magna*. Many of these species were identified in urine for the first time, their role in urolithiasis has thus not been explored. Clinically, most patients present with unilateral stones. We assumed that there might be some protective bacteria in the non-stone side which inhibit causative bacteria colonization to maintain a balanced urobiome and create an anti-lithogenic environment.

There were some limitations in our study. First, the sample size was relatively small, and a larger sample size was required to validate our results. Second, our study was not powered to detect a significant association between two clusters and clinical characteristics. It would be valuable if stone composition showed different distributions between Stone1 and Stone2. However, it is a pity that no significant differences were observed in stone composition between the two clusters. This might be attributed to an insufficient number of samples and missing data on chemical composition analysis for about 40% of patients. Third, 2bRAD-M is unable to predict the potential functional pathways of the urobiome. Thus, our study had missing data for functional information on the microbial communities. Finally, our study was based on descriptive microbiome research, in which is difficult to determine the causal relationship between the urobiome and urolithiasis. Experimental and longitudinal follow-up studies are needed to investigate whether bacteria are initiators of or bystanders in stone formation.

## 5. Conclusions

In summary, we found that the renal pelvis urobiome in the stone side of unilateral stone formers was separated into two clusters, namely Stone1 and Stone2. Stone2 exhibited greater microbial richness and diversity compared to Stone1. The genera, *Cupriavidus* and *Sphingomonas*, were enriched in Stone1, while *Acinetobacter* and *Pseudomonas* were enriched in Stone2. Many differential species were identified between Stone1 and Stone2. A random forest model achieved a powerful diagnostic potential to distinguish the two clusters. There might be different mechanisms through which microbiota contribute to stone formation in two clusters, and our findings might assist in the selection of suitable medical treatments for urolithiasis.

## Figures and Tables

**Table 1:** Demographic and clinical characteristics of two clusters.

Parameter	Stone1 (*n* = 11)	Stone2 (*n* = 16)	*p*-Value
Age	51.09 ± 18.26	48.13 ± 14.55	0.643
Gender			0.692
Male	8 (72.7%)	10 (62.5%)	
Female	3 (27.3%)	6 (37.5%)	
Body mass index (kg/m^2^)	24.34 ± 3.53	23.78 ± 1.79	0.575
Stone side			0.097
Left	5 (45.5%)	13 (81.3%)	
Right	6 (54.5%)	3 (18.8%)	
Stone count			1.000
Single	7 (63.6%)	9 (56.3%)	
Multiple	4 (36.4%)	7 (43.8%)	
Stone composition			0.496
CaOx	3 (27.3%)	4 (25%)	
CaOx + CaP	1 (9.1%)	5 (31.3%)	
Uric acid	2 (18.2%)	1 (6.3%)	
Not available	5 (45.5%)	6 (37.5%)	
Comorbidities			0.242
Hypertension	1 (9.1%)	5 (31.3%)	
Diabetes	0 (0%)	1 (6.3%)	

## Data Availability

Sequencing data from this study have been deposited in the GenBank Sequence Read Archive under accession number PRJNA843211 (https://www.ncbi.nlm.nih.gov/bioproject/PRJNA843211/) accessed on 21 July 2021.

## Supplementary Materials

The following supporting information can be downloaded at: https://www.mdpi.com/article/10.3390/microorganisms11092276/s1, Figure S1. Clustering of the renal pelvis urine samples. 6 distance algorithms, including Binary Jaccard (A), Bray-Curtis (B), Euclidean (C), JSD (D), L1 (E), and L2 (F) distance were applied to cluster the samples. Table S1. Comparison of average relative abundance of pelvis urinary microbiome between Stone1 and Stone2 at the genus and species levels.

## References

[1] Zeng G., Mai Z., Xia S., Wang Z., Zhang K., Wang L., Long Y., Ma J., Li Y., Wan S.P. (2017). Prevalence of kidney stones in China: An ultrasonography based cross-sectional study. BJU Int..

[2] D’Costa M.R., Haley W.E., Mara K.C., Enders F.T., Vrtiska T.J., Pais V.M., Jacobsen S.J., McCollough C.H., Lieske J.C., Rule A.D. (2019). Symptomatic and Radiographic Manifestations of Kidney Stone Recurrence and Their Prediction by Risk Factors: A Prospective Cohort Study. J. Am. Soc. Nephrol..

[3] Thongprayoon C., Krambeck A.E., Rule A.D. (2020). Determining the true burden of kidney stone disease. Nat. Rev. Nephrol..

[4] Baumann J.M., Affolter B. (2014). From crystalluria to kidney stones, some physicochemical aspects of calcium nephrolithiasis. World J. Nephrol..

[5] Hong S.Y., Xia Q.D., Yang Y.Y., Li C., Zhang J.Q., Xu J.Z., Qin B.L., Xun Y., Wang S.G. (2022). The role of microbiome: A novel insight into urolithiasis. Crit. Rev. Microbiol..

[6] Whiteside S.A., Razvi H., Dave S., Reid G., Burton J.P. (2015). The microbiome of the urinary tract--a role beyond infection. Nat. Rev. Urol..

[7] Karam A., Mjaess G., Albisinni S., El Daccache Y., Farah M., Daou S., Kazzi H., Hassoun R., Bou Kheir G., Aoun F. (2022). Uncovering the role of urinary microbiota in urological tumors: A systematic review of literature. World J. Urol..

[8] Xie J., Huang J.S., Huang X.J., Peng J.M., Yu Z., Yuan Y.Q., Xiao K.F., Guo J.N. (2020). Profiling the urinary microbiome in men with calcium-based kidney stones. BMC Microbiol..

[9] Liu F., Zhang N., Jiang P., Zhai Q., Li C., Yu D., Wu Y., Zhang Y., Lv L., Xu X. (2020). Characteristics of the urinary microbiome in kidney stone patients with hypertension. J. Transl. Med..

[10] Dornbier R.A., Bajic P., Van Kuiken M., Jardaneh A., Lin H., Gao X., Knudsen B., Dong Q., Wolfe A.J., Schwaderer A.L. (2020). The microbiome of calcium-based urinary stones. Urolithiasis.

[11] Shen C., Zhu Q., Dong F., Wang W., Fan B., Li K., Chen J., Hu S., He Z., Li X. (2021). Identifying Two Novel Clusters in Calcium Oxalate Stones With Urinary Tract Infection Using 16S rDNA Sequencing. Front. Cell. Infect. Microbiol..

[12] Sun Z., Huang S., Zhu P., Tzehau L., Zhao H., Lv J., Zhang R., Zhou L., Niu Q., Wang X. (2022). Species-resolved sequencing of low-biomass or degraded microbiomes using 2bRAD-M. Genome Biol..

[13] Oksanen J., Blanchet F.G., Kindt R., Legendre P., Minchin P., O’Hara B., Simpson G., Solymos P., Stevens H., Wagner H. (2015). Vegan: Community Ecology Package. R Package Version 2.2-1. Agric. Sci..

[14] Segata N., Izard J., Waldron L., Gevers D., Miropolsky L., Garrett W.S., Huttenhower C. (2011). Metagenomic biomarker discovery and explanation. Genome Biol..

[15] Liaw A., Wiener M. (2001). Classification and Regression by RandomForest. Forest.

[16] Robin X., Turck N., Hainard A., Tiberti N., Lisacek F., Sanchez J.C., Müller M. (2011). pROC: An open-source package for R and S+ to analyze and compare ROC curves. BMC Bioinform..

[17] Ren Z., Li A., Jiang J., Zhou L., Yu Z., Lu H., Xie H., Chen X., Shao L., Zhang R. (2019). Gut microbiome analysis as a tool towards targeted non-invasive biomarkers for early hepatocellular carcinoma. Gut.

[18] Daskalakis M.I., Magoulas A., Kotoulas G., Catsikis I., Bakolas A., Karageorgis A.P., Mavridou A., Doulia D., Rigas F. (2013). Pseudomonas, Pantoea and Cupriavidus isolates induce calcium carbonate precipitation for biorestoration of ornamental stone. J. Appl. Microbiol..

[19] Daskalakis M.I., Magoulas A., Kotoulas G., Katsikis I., Bakolas A., Karageorgis A.P., Mavridou A., Doulia D., Rigas F. (2014). Cupriavidus metallidurans biomineralization ability and its application as a bioconsolidation enhancer for ornamental marble stone. Appl. Microbiol. Biotechnol..

[20] Liu J., Su J., Ali A., Wang Z., Zhang R. (2022). Potential of a novel facultative anaerobic denitrifying Cupriavidus sp. W12 to remove fluoride and calcium through calcium bioprecipitation. J. Hazard. Mater..

[21] Randall A. (1937). THE ORIGIN AND GROWTH OF RENAL CALCULI. Ann. Surg..

[22] Miller N.L., Gillen D.L., Williams J.C., Evan A.P., Bledsoe S.B., Coe F.L., Worcester E.M., Matlaga B.R., Munch L.C., Lingeman J.E. (2009). A formal test of the hypothesis that idiopathic calcium oxalate stones grow on Randall’s plaque. BJU Int..

[23] Gay C., Letavernier E., Verpont M.C., Walls M., Bazin D., Daudon M., Nassif N., Stéphan O., de Frutos M. (2020). Nanoscale Analysis of Randall’s Plaques by Electron Energy Loss Spectromicroscopy: Insight in Early Biomineral Formation in Human Kidney. ACS Nano.

[24] Liu F., Zhang N., Wu Y., Jiang P., Jiang T., Wang Y., Zhang Y., Zhai Q., Zou Y., Feng N. (2020). The pelvis urinary microbiome in patients with kidney stones and clinical associations. BMC Microbiol..

[25] Tang R., Jiang Y., Tan A., Ye J., Xian X., Xie Y., Wang Q., Yao Z., Mo Z. (2018). 16S rRNA gene sequencing reveals altered composition of gut microbiota in individuals with kidney stones. Urolithiasis.

[26] Barr-Beare E., Saxena V., Hilt E.E., Thomas-White K., Schober M., Li B., Becknell B., Hains D.S., Wolfe A.J., Schwaderer A.L. (2015). The Interaction between Enterobacteriaceae and Calcium Oxalate Deposits. PLoS ONE.

[27] Fishman M.R., Giglio K., Fay D., Filiatrault M.J. (2018). Physiological and genetic characterization of calcium phosphate precipitation by Pseudomonas species. Sci. Rep..

[28] Safari A., Habimana O., Allen A., Casey E. (2014). The significance of calcium ions on Pseudomonas fluorescens biofilms—A structural and mechanical study. Biofouling.

[29] Chutipongtanate S., Sutthimethakorn S., Chiangjong W., Thongboonkerd V. (2013). Bacteria can promote calcium oxalate crystal growth and aggregation. J. Biol. Inorg. Chem..

[30] Kanlaya R., Naruepantawart O., Thongboonkerd V. (2019). Flagellum Is Responsible for Promoting Effects of Viable Escherichia coli on Calcium Oxalate Crystallization, Crystal Growth, and Crystal Aggregation. Front. Microbiol..

[31] Djojodimedjo T., Soebadi D.M., Soetjipto (2013). Escherichia coli infection induces mucosal damage and expression of proteins promoting urinary stone formation. Urolithiasis.

[32] Domrongkitchaiporn S., Stitchantrakul W., Kochakarn W. (2006). Causes of hypocitraturia in recurrent calcium stone formers: Focusing on urinary potassium excretion. Am. J. Kidney Dis..

