# Supplementary material for: Identification of Two Clusters in Renal Pelvis Urobiome of Unilateral Stone Formers Using 2bRAD-M
Source: Microorganisms. 2023 Sep 10;11(9):2276. doi: 10.3390/microorganisms11092276 (PMC10534321; doi:10.3390/microorganisms11092276)
Supplement: Supplementary file 1 [file microorganisms-11-02276-s001.zip › Table S1.pdf]

Table S1 - Comparison of average relative abundance of pelvis urinary microbiome between Stone1 and Stone2 at the genus and species levels

Taxa		Average relative abundance (%)		
		Stone1	Stone2	P-value
genus	Acinetobacter	8.000	62.129	0.000
	Cupriavidus	57.098	3.593	0.000
	Pseudomonas	2.191	12.402	0.000
	Sphingomonas	21.800	0.704	0.000
	Moraxella	1.007	5.969	0.000
	Stenotrophomonas	0.651	8.429	0.000
	Achromobacter	0.000	0.069	0.001
	Clavispora	0.040	0.238	0.001
	Meyerozyma	0.048	0.174	0.003
	Alcaligenes	0.356	0.006	0.004
	Sphingobium	0.019	0.232	0.005
	Jeongeupia	0.034	0	0.022
	Brevundimonas	0.094	0.585	0.028
species	Cupriavidus pauculus	54.367	3.479	0.000
	Acinetobacter sp_CIP_110321	2.924	17.440	0.000
	Cupriavidus metallidurans	2.675	0.103	0.000
	Sphingomonas paucimobilis	20.182	0.360	0.000
	Moraxella osloensis	1.007	5.969	0.000
	Acinetobacter junii	2.028	28.472	0.000
	Acinetobacter sp_MN12	0.154	3.036	0.000
	Stenotrophomonas sp_LMG_10879	0.058	0.962	0.000
	Pseudomonas fluorescens	1.200	6.304	0.000
	Acinetobacter ursingii	2.554	8.787	0.000
	Acinetobacter johnsonii	0.096	3.744	0.000
	Stenotrophomonas maltophilia	0.592	7.079	0.000
	Stenotrophomonas rhizophila	0.000	0.102	0.000
	Pseudomonas putida	0.016	1.034	0.001
	Xanthomonas arboricola	0.000	0.117	0.001
	Clavispora lusitaniae	0.040	0.238	0.001
	Cupriavidus taiwanensis	0.027	0.000	0.002
	Meyerozyma guilliermondii	0.048	0.174	0.003
	Alcaligenes faecalis	0.356	0.006	0.004
	Sphingobium yanoikuyae	0.019	0.227	0.006
	Sphingomonas koreensis	0.307	0.000	0.007
	Pseudomonas koreensis	0.000	0.292	0.008
	Achromobacter sp_ATCC31444	0.000	0.042	0.009
	Sphingomonas sp_S_NIH_Pt1_0416	1.227	0.000	0.016
	Brevundimonas sp_DS20	0.032	0.370	0.018
	Pseudomonas sp_HMWF006	0.012	0.149	0.019
	Jeongeupia sp_USM3	0.034	0.000	0.022

Pseudomonas marginalis	0.000	0.182	0.028
Acinetobacter colistiniresistens	0.000	0.222	0.028
Pseudomonas sp_NBRC_111144	0.000	0.612	0.031
Pseudomonas syringae_pv_syringae_B728a	0.000	0.259	0.041
Pseudomonas sp_GM60	0.002	0.070	0.045
