# Supplementary figures and images for: Identification of Two Clusters in Renal Pelvis Urobiome of Unilateral Stone Formers Using 2bRAD-M
Source: Microorganisms. 2023 Sep 10;11(9):2276. doi: 10.3390/microorganisms11092276 (PMC10534321; doi:10.3390/microorganisms11092276)

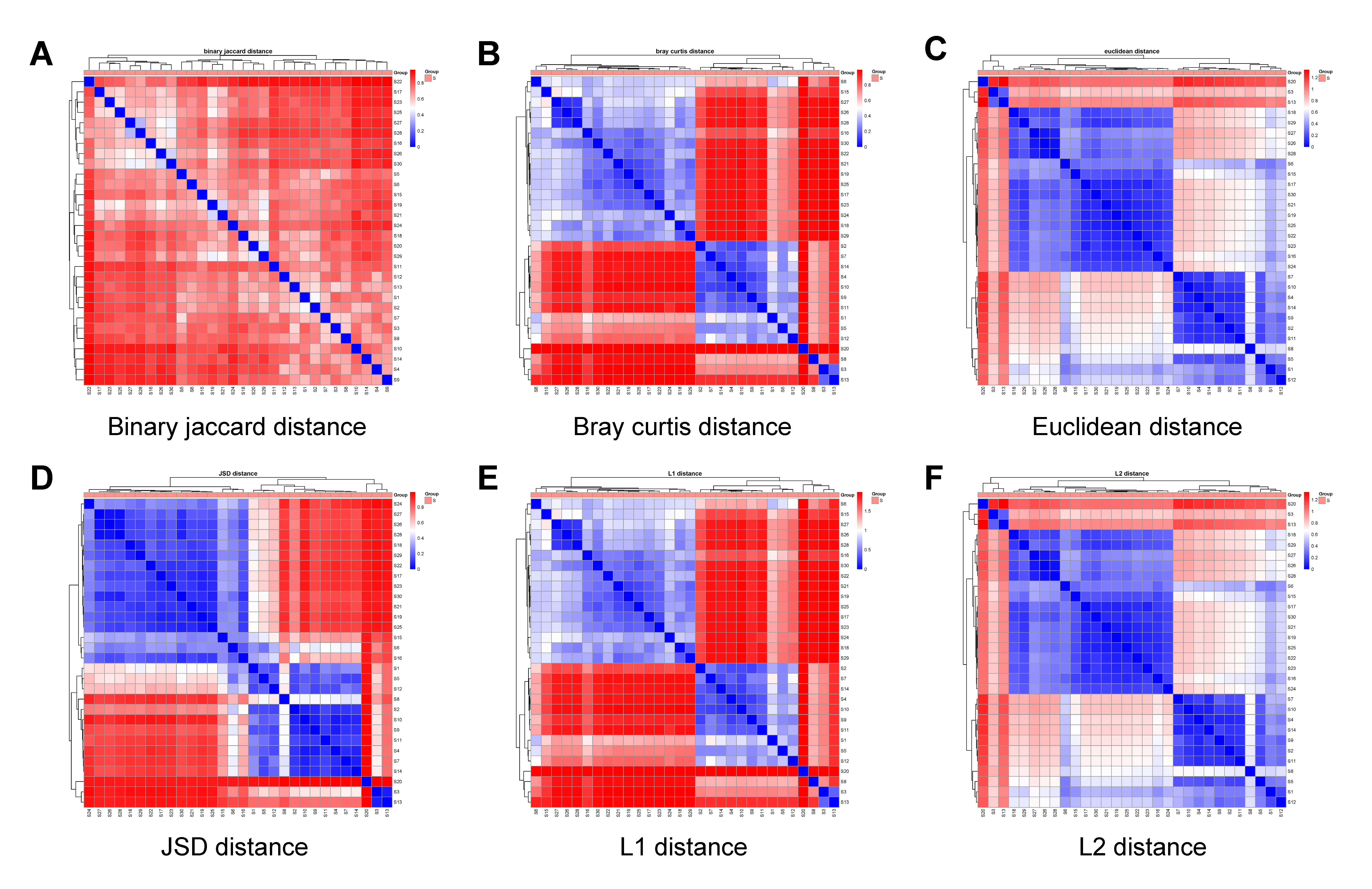

Supplement: Supplementary file 1 [file microorganisms-11-02276-s001.zip › Figure S1.tif]
